# F-18 fluorodeoxyglucose positron emission tomography for differential diagnosis of pancreatic tumors

**DOI:** 10.1186/s40064-015-0938-2

**Published:** 2015-03-31

**Authors:** Masato Yoshioka, Hiroshi Uchinami, Go Watanabe, Tsutomu Sato, Satoshi Shibata, Makoto Kume, Koichi Ishiyama, Satoshi Takahashi, Manabu Hashimoto, Yuzo Yamamoto

**Affiliations:** Department of Gastroenterological Surgery, Akita University Graduate School of Medicine, 1-1-1 Hondo, Akita, 010-8543 Japan; Department of Surgery, Akita City Hospital, Akita, 010-0933 Japan; Department of Surgery, Honjo Daiichi Hospital, Honjo, Akita, 015-8567 Japan; Department of Surgery, Murakami Memorial Hospital, Asahi University, Gifu, 500-8523 Japan; Department of Radiology, Akita University Graduate School of Medicine, 1-1-1 Hondo, Akita, 010-8543 Japan

**Keywords:** FDG-PET, SUV, Cut-off, IPMN, Non-ductal pancreatic cancer

## Abstract

Positron emission tomography with 2-deoxy-2-[^18^F]fluoro-D-glucose (FDG-PET) has been proven useful for differentiating pancreatic ductal cancer from mass-forming chronic pancreatitis. However, there are particular pancreatic tumors having various grades of malignancy such as intraductal papillary mucinous neoplasm (IPMN) or pancreatic neuroendocrine tumor. We examined whether the cut-off value of maximum standardized uptake value (SUV_max_) determined by pancreatic ductal cancers is also applicable for other pancreatic tumors.

One hundred thirty six patients with pancreatic tumors underwent FDG-PET imaging. We first analyzed the cut-off value to differentiate pancreatic ductal cancers from mass-forming chronic pancreatitis. Secondly, we determined the cut-off value between malignant IPMN and benign IPMN. Thirdly, we computed a cut-off value between malignant pancreatic tumors and benign tumors irrespective of tumor type.

The optimal cut-off value to differentiate ductal cancers from mass-forming chronic pancreatitis was 2.5. The optimal cut-off value for differentiating malignant IPMN from benign IPMN was also 2.5, similar to that of reported studies. In all types of pancreatic tumors, the cut-off value was also 2.5. The accuracy for detecting malignancy was 93.4% for all tumors.

In the FDG-PET study for pancreatic tumors, an SUV_max_ of 2.5 would be justified as a cut-off value to differentiate malignant lesions.

## Background

Owing the progress of imaging modalities such as abdominal ultrasonography (US) and computed tomography (CT), accuracy in the diagnosis of pancreatic tumors has improved over the last decade. In pancreatic tumors, however, there are a variety of tumors such as ductal neoplasms, inflammatory and fibrotic tumors, and cystic tumors with malignant potentials of various degrees. Among them, intraductal papillary mucinous neoplasm (IPMN) and pancreatic neuroendocrine tumor (pNET) are the tumors in which it is difficult to distinguish malignant ones from benign ones preoperatively.

Positron emission tomography using 2-deoxy-2-[^18^F]fluoro-D-glucose (FDG-PET) is a noninvasive, useful imaging modality. Theoretical background is based on the difference of cellular glucose metabolism (Rempel et al. 1996). FDG-PET is reported as a valuable measure for diagnosing and staging various kinds of cancers (Delbeke 1999; Berberat et al. 1999; Kubota et al. 1990; Ishizu et al. 1994; Bares et al. 1994; Wahl et al. 1991; Jansson et al. 1995; Yoshioka et al. 2004). The usefulness of FDG-PET in differentiating pancreatic ductal cancer from mass-forming chronic pancreatitis has been reported. Maximum standardized uptake value (SUV_max_) is a common parameter for evaluating the uptake by a mass lesion semi-quantitatively. This value is defined as the radioactivity of the tissue divided by the total radioactivity of the probe isotope injected per body weight (Sadato et al. 1998). The cut-off values of SUV_max_ in differentiating pancreatic ductal cancer from mass-forming chronic pancreatitis have already been reported. Recommendable cut-off values for detecting malignancy of IPMN were previously reported in several studies (Tomimaru et al. 2010; Takanami et al. 2011; Hong et al. 2010), but the patient number of each study was very small as 29, 16 and 31 patients, respectively.

In this study, as reported in other studies, we first analyzed the cut-off value of SUV_max_ to differentiate patients with pancreatic ductal cancer from mass-forming chronic pancreatitis in our patient series. Secondly, similar analysis was done between the malignant and benign IPMNs, and the obtained cut-off value was compared with those in 3 published studies. Thirdly, we computed a cut-off value to differentiate the malignant pancreatic tumors from the benign tumors irrespective of tumor type including pNET, and examined whether this cut-off value was applicable for detecting pancreatic malignancy in general.

## Results

**Figure 1:**
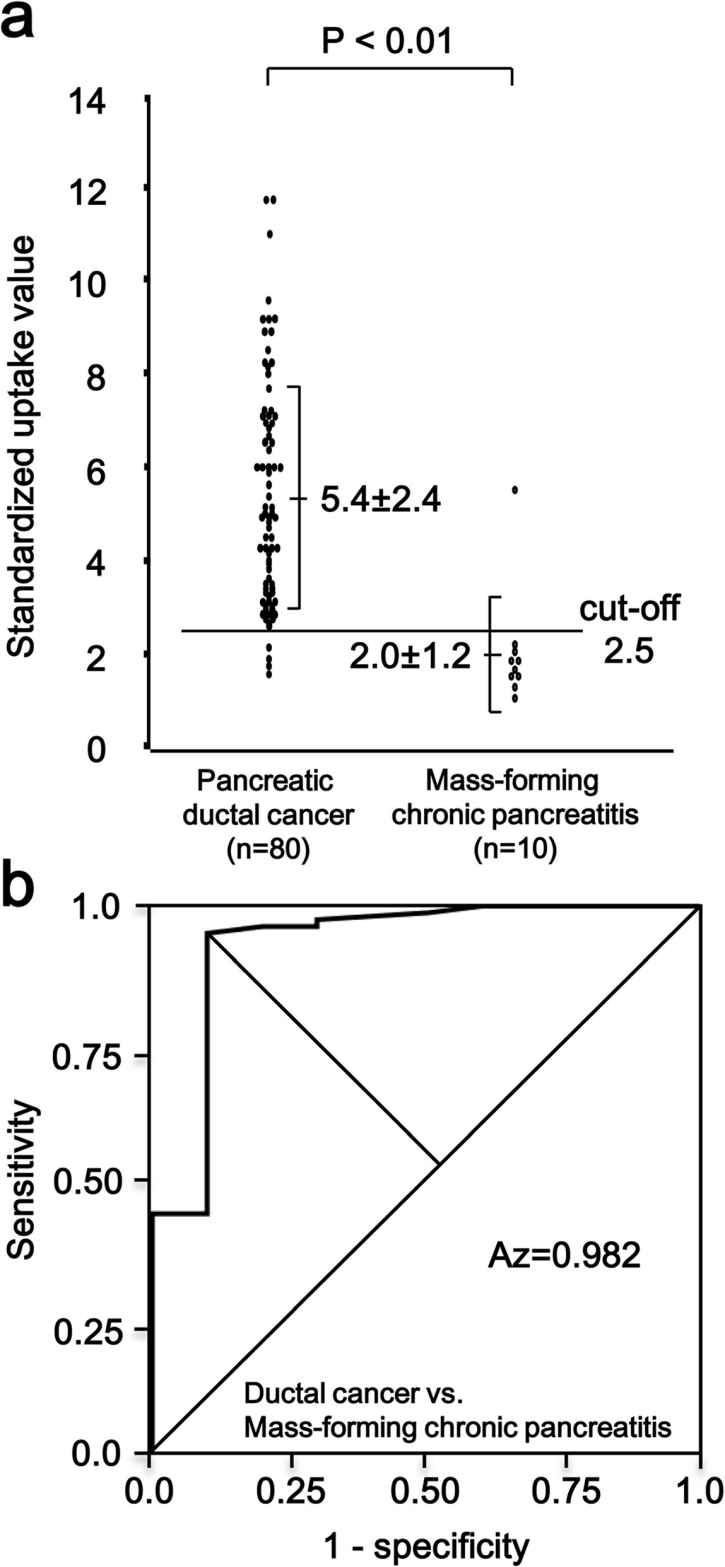
**Maximum standardized uptake value (SUV**_**max**_ **) in pancreatic ductal cancer and mass-forming chronic pancreatitis. (a)** There is a significant difference in SUV_max_ between pancreatic ductal cancer and mass-forming chronic pancreatitis non-parametrically, *P* < 0.01. The number in each column is the mean SUV_max_ ± standard deviation. **(b)** Receiver operating characteristic curve in pancreatic ductal cancer and mass-forming chronic pancreatitis. The area under the curve is 0.982.

Table 1 shows the number of patients and the SUV_max_ in the patients of each disease. The SUV_max_ in pancreatic ductal cancer and mass-forming chronic pancreatitis are illustrated in Figure 1a. There was a significant difference between these groups (*P* < 0.01). Figure 1b demonstrates the ROC analysis of SUVs between ductal cancer and mass-forming chronic pancreatitis. The optimal SUV_max_ to differentiate ductal cancer from mass-forming chronic pancreatitis was 2.5, which was determined as the point of the curve farthest from the chance line. The areas divided by the curve were used to verify the performance of the analysis. The area under the curve was 0.982, which showed this analysis was appropriate. When an SUV_max_ of 2.5 was set as the cut-off value, only 4 of the ductal cancer lesions (SUV_max_ = 2.2, 1.9, 1.8 and 1.6, respectively) had an SUV_max_ below the cut-off value (false-negative). On the other hand, only one mass-forming chronic pancreatitis lesion (SUV_max_ = 5.3) had an SUV_max_ above the cut-off value (false-positive). This patient has IgG4-related autoimmune pancreatitis (AIP). To summarize, when the cut-off value was set at 2.5, sensitivity, specificity, positive predictive value, negative predictive value, and accuracy rate between ductal cancer and mass-forming chronic pancreatitis were shown in Table 2.

**Table 1:** **The mean and range of maximum SUV in patients of each disease**

**Disease**	**Malignant/Benign**	**No. of Patients**	**Mean SUV**_**max**_ **(Range)**
Pancreatic ductal cancer	Malignant	80	5.4 (1.6-11.8)
Mass-forming chronic pancreatitis	Benign	10	2.0 (1.2-5.3)
Intraductal papillary mucinous carcinoma	Malignant	18	5.5 (1.5-13.8)
Intraductal papillary mucinous neoplasm	Benign	12	1.7 (1.1-2.3)
Neuroendocrine carcinoma	Malignant	4	
Non-functioning islet cell carcinoma		4	9.0 (3.3-13.8)
Neuroendocrine tumor	Benign	12	
Insulinoma		7	1.6 (1.1-2.0)
Glucagonoma		2	1.9 (1.8-2.0)
Gastrinoma		1	1.8
Non-functioning islet cell tumor		2	2.2 (1.9-2.4)

SUV: standardized uptake value, SUV_mav_: maximum SUV.

**Table 2:** **The efficacy of the cut-off value in differential diagnosis**

	**Cut-off (SUV**_**max**_ **)**	**Az value**	**SEN (%)**	**SPE (%)**	**PPV (%)**	**NPV (%)**	**ACC (%)**
Pancreatic ductal cancer vs. Mass-forming chronic pancreatitis	2.5	0.982	95.0	90.0	98.7	69.2	94.4
Malignant IPMN vs. Benign IPMN	2.5	0.933	77.8	100	100	75.0	86.7
All malignant tumors vs. All benign tumors	2.5	0.957	92.2	97.1	98.9	80.1	93.4

SUV_max_: maximum standardized uptake value, Az: ROC-area index, SEN: sensitivity, SPE: specificity, PPV: positive predictive value, NPV: negative predictive value, ACC: accuracy, IPMN: intraductal papillary mucinous neoplasm.

**Figure 2:**
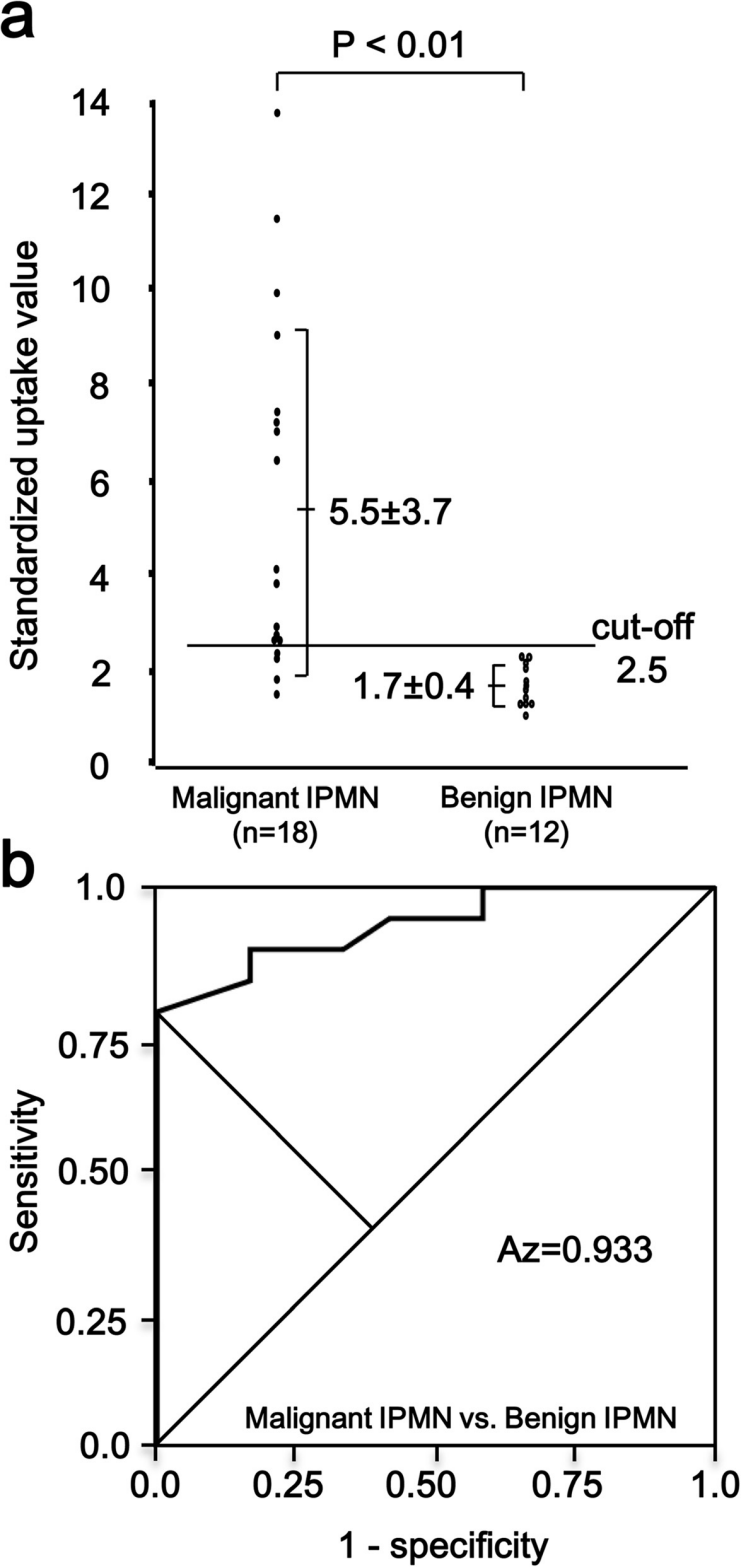
**Maximum standardized uptake value (SUV**_**max**_ **) in malignant intraductal papillary mucinous neoplasm (IPMN) and benign IPMN. (a)** There is a significant difference in SUV_max_ between malignant IPMN and benign IPMN non-parametrically, *P* < 0.01. The number in each column is the mean SUV_max_ ± standard deviation. **(b)** Receiver operating characteristic curve in malignant IPMN and benign IPMN. The area under the curve is 0.933.

The SUV_max_ in malignant IPMN (IPMC) and benign IPMN are illustrated in Figure 2a. There was a significant difference between these groups (*P* < 0.01). Figure 2b illustrates the ROC analysis between IPMC and benign IPMN. The optimal SUV_max_ to differentiate IPMC from benign IPMN was also 2.5. The area under the curve was 0.933. When an SUV_max_ of 2.5 was set as the cut-off value, 4 of the malignant IPMNs (SUV_max_ = 2.3, 2.2, 1.8 and 1.5, each) showed an SUV_max_ below the cut-off value (false-negative). These 4 patients were all carcinoma in situ (CIS). There was no patient showing false-positive in the benign IPMN group.

**Figure 3:**
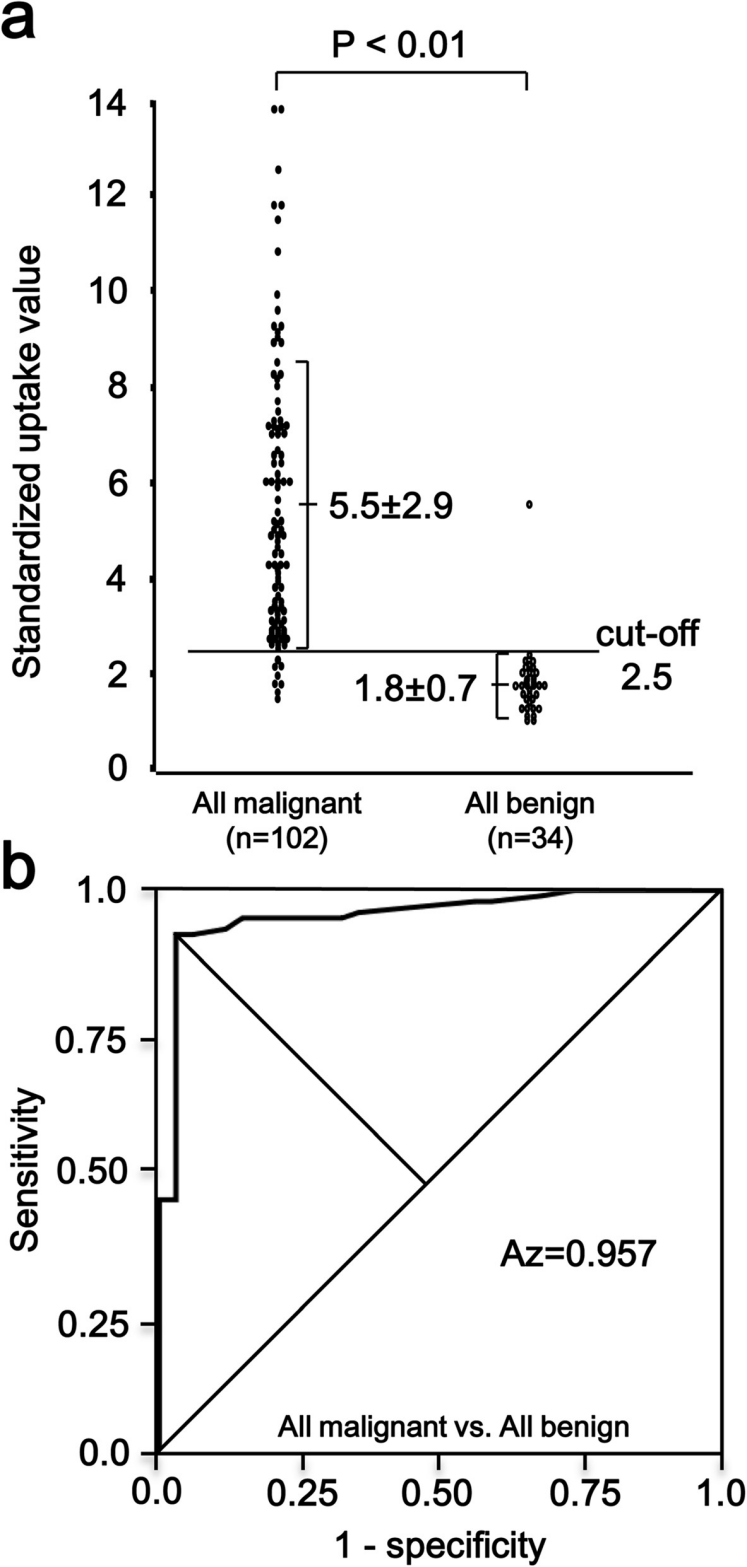
**Maximum standardized uptake value (SUV**_**max**_ **) in all malignant tumors and all benign tumors. (a)** There is a significant difference in SUV_max_ between all malignant tumors and all benign tumors non-parametrically, *P* < 0.01. The number in each column is the mean SUV_max_ ± standard deviation. **(b)** Receiver operating characteristic curve in all malignant tumors and all benign tumors. The area under the curve is 0.957.

Since the cut-off value to differentiate ductal cancer from mass-forming chronic pancreatitis was the same as the cut-off value to differentiate IPMC from benign IPMN (that is 2.5), we unified the data of all malignant tumors and all benign tumors including pNETs—pNETs were not suitable for independent analysis due to their small number of cases—and re-computed a cut-off value to discriminate between malignant and benign tumors of the pancreas. The SUV_max_ in all malignant tumors and all benign tumors was illustrated in Figure 3a. There was a significant difference between these groups (*P* < 0.01). The optimal SUV_max_ to differentiate all malignant tumor lesions from all benign tumor lesions was exactly 2.5 (Figure 3b). The area under the curve was 0.957. When an SUV_max_ of 2.5 was set as the cut-off value, only 8 of the malignant tumors (4 ductal cancers and 4 IPMCs [CIS]) had an SUV_max_ below the cut-off value (false-negative). On the other hand, in all benign tumors, only one patient (IgG4-related AIP) showed false-positive. Sensitivity, specificity and accuracy rate were 92.2%, 97.1% and 93.4%, respectively (Table 2). As to pNET, SUV_max_ of four malignant non-functioning islet cell tumors were all above this cut-off value of 2.5 (SUV = 13.8, 12.5, 6.2 and 3.3); and all of 12 benign pNETs showed SUV_max_ below this cut-off value, although the case number is limited.

## Discussion

In the differential diagnosis between pancreatic ductal cancer and mass-forming chronic pancreatitis, high accuracy of FDG-PET—greater than 85%—has already been noted (Inokuma et al. 1995; Nakamoto et al. 1999; Imdahl et al. 1999; Nitzsche et al. 2002). Usually, increased FDG uptake by the tumor is visually assessed in comparison with that by surrounding tissues. This classical method is, however, sometimes not useful for differential diagnosis, especially when the tumors are not sufficiently large. In contrast, an SUV_max_ offering a semi-quantitative analysis is more advantageous. In this study, the cut-off value of SUV_max_ for differentiating pancreatic ductal cancer from mass-forming chronic pancreatitis was 2.5. This value was comparable with those obtained in other studies (Nakamoto et al. 2000; Delbeke et al. 1999), indicating that a cut-off value of 2.5 for this purpose was credible. On the other hand, there were several studies dealing about the usefulness of FDG-PET for differential diagnosis between malignant and benign IPMN (Tomimaru et al. 2010; Takanami et al. 2011; Hong et al. 2010; Sperti et al. 2001, 2005, 2007; Mansour et al. 2006; Tann et al. 2007; Pedrazzoli et al. 2011). But some studies, in addition to IPMNs, included other cystic tumors, ductal cancer and histologically undetermined tumors as well (Sperti et al. 2001, 2005, 2007; Mansour et al. 2006; Tann et al. 2007; Pedrazzoli et al. 2011). There were only 3 studies that calculated the accuracy within genuine IPMNs (Tomimaru et al. 2010; Takanami et al. 2011; Hong et al. 2010). Tomimaru et al. first reported the usefulness of SUV_max_ in differentiating malignant and benign IPMNs (Tomimaru et al. 2010). In their study, patient number was 14 with malignancy and 15 with benign IPMNs. They set the best cut-off value as 2.5 with a high accuracy of 96%. Takanami et al. reported the second study (Takanami et al. 2011). In their report, there were 9 malignant IPMNs and 7 benign IPMNs (total 16 patients), and a cut-off value of 2.3 was the best with a high accuracy of 87.5%. Thirdly, Hong et al. described that the diagnostic accuracy of a cut-off value using SUV_max_ 2.5 was as high as 96% in 31 patients (Hong et al. 2010). Regrettably, they merely adopted this cut-off value from other reports that dealt with miscellaneous types of cystic tumors (Sperti et al. 2005, 2007; Mansour et al. 2006; Tann et al. 2007) and skipped the process of statistical estimation. They gave no explanation as to why they used an SUV_max_ of 2.5 as a cut-off value. In contrast, the former two reports are distinguishing because they determined their cut-off values by ROC analysis. However, even in these reports, the numbers of patient were only 29 and 16, respectively. Since the incidence of IPMN is relatively small in comparison with ductal cancer, there is a limitation in the study performed by a single institution. Indeed, in our study also, the patient number is 30 and still small. Nevertheless, the cut-off values independently determined by these three studies were consistent. Hence, the cut-off value 2.5 seems to be acceptable for differentiating IPMC from benign IPMN.

Meanwhile, in regard to pNET, there is no study dealing with the cut-off value of SUV_max_ in differentiating malignancy because patient number is further small. However, interestingly, PET scan in our study detected high FDG uptake above 2.5 in all 4 patients with non-functioning islet cell carcinoma, and below 2.5 in all benign pNETs. Although we cannot draw a definitive conclusion, it is likely that cut-off value of 2.5 would be useful even for pNET. Further study is intriguing.

We reviewed 8 false-negative lesions in malignancies (4 ductal cancers and 4 IPMCs) and one false-positive lesion in mass-forming chronic pancreatitis in detail. We cannot explain the reason why 4 patients (5.0%) having ductal cancer showed false-negative (SUV_max_ = 2.2, 1.9, 1.8 and 1.6, each) because there was no difference between these 4 tumors and others concerning the tumor size and histological differentiation. Four of 18 patients (22.2%) having IPMC showed false-negative (SUV_max_ = 2.3, 2.2, 1.8 and 1.5, each). These tumors were all CIS. To detect the CIS preoperatively is extremely difficult, and FDG-PET would regard CIS as benign lesion. There was only one false-positive lesion in mass-forming chronic pancreatitis. This case had IgG4-related AIP. If serum IgG4 level or other markers that indicate autoimmunity had been elevated preoperatively, we could suspect IgG4-related AIP in the first place. However, in our patient, these markers were all within normal limits. In this way, there are patients showing that serum IgG4 and other markers are within normal limits. In these cases, preoperative diagnosis is not easy. As to FDG-PET for IgG4-related AIP, some case reports are published, but the study of FDG-PET in differential diagnosis of IgG4-related AIP has not been performed yet because patient number is extremely limited for submitting analysis. Kamisawa et al. compared the SUV_max_ of autoimmune pancreatitis and pancreatic cancer (Kamisawa et al. 2010). They showed that there was no significant difference between the SUV_max_ of 10 AIP patients and that of 14 pancreatic cancer patients. This is only one study and preliminary, but the result would suggest the difficulty in differential diagnosis of AIP using FDG-PET. Further prospective study with a larger number of cases is encouraged.

In the past 15 years, new technologies have been developed. With regard to tracers, ^18^F labelled deoxy-fluorothymidine has been tested for detecting pancreatic cancers (Herrmann et al. 2012). For pNET, somatostatin analogues (TETA-Y3-TATE, DOTA-Tyr3-octreotide) have been labelled with ^64^Cu or ^68^Ga (Lewis et al. 1999; Gabriel et al. 2007). For detecting insulinoma, specific probe targeting glucagon-like peptide-1 receptor has been developed (Brom et al. 2010; Wild et al. 2010, Eriksson et al. 2014). As a new imaging modality, PET-MRI was also developed and tested for detecting the gastroenteropancreatic NET (Beiderwellen et al. 2013). However, these new tracers and machines have been used in trial only at some limited institutions, and thus their usefulness is still under evaluation. It will take a little time before these methods become widespread in the hospitals where ordinary patients will access. The most useful strategy at present seems to be FDG-PET/CT.

## Conclusions

This study suggested a good potential of FDG-PET in differentiating malignancy also in pancreatic tumors other than ductal cancer. Calculation of SUV_max_ would be greatly helpful for differential diagnosis when the malignancy is uncertain in pancreatic lesions despite the full use of conventional imaging modalities. It was indicated that an SUV_max_ of 2.5 would be justified as a cut-off value for malignant pancreatic tumors in general, as it is for ductal cancer.

## Materials and methods

### Patients

From October 2001 to December 2014, 136 patients (76 males and 60 females; mean age, 66 years old; age range 26-88 years old) were diagnosed as having pancreatic tumors by US, CT, and magnetic resonance imaging (MRI) in our department. All of them underwent FDG-PET. The diagnosis was histologically proven and the final diagnoses were 80 pancreatic ductal cancers, 10 mass-forming chronic pancreatitis, 18 intraductal papillary mucinous carcinoma (IPMC), 12 benign IPMN, 4 malignant pNETs (non-functioning islet cell carcinoma) and 12 benign pNETs (7 insulinoma, 2 glucagonoma, 1 gastrinoma and 2 non-functioning islet cell tumor). The research protocol of this study was approved by the Ethics Committee of Akita University Graduate School of Medicine (No.1223), and each patient gave written informed consent before enrollment in the study.

### FDG-PET

FDG-PET studies were performed either with Headtome V (Shimadzu Co., Kyoto, Japan) or with PET-CT scanner of Discovery ST Elite 16 (GE healthcare, Milwaukee, WI, USA). Patients having pancreatic cancer and/or chronic pancreatitis often show hyperglycemia. Because the uptake of ^18^F-FDG by the tumors is reduced in hyperglycemic status, the guideline for FDG-PET/CT recommends that examination be performed under the blood glucose level lower than 150-200 mg/dl (Delbeke et al. 2006). All patients enrolled in this study fasted for 6 hours before examination and were checked for their blood glucose level just before examination. When the blood glucose level was greater than 150 mg/dl, we rescheduled the examination. The images were acquired first at 60 minutes after ^18^F-FDG of approximately 185 MBq injection. CT scanning was performed with 120 kV tube voltage and Auto mA. Images were reconstructed with a slice thickness of 3.75 mm. Neither intravenous nor oral contrast materials were used. Acquisitions were performed in 3-dimensional mode, 3 min/bed position. Data were reconstructed using VUE Point Plus; ordinary Poisson OSEM (Ordered Subset Expectation Maximization) with 10 subsets and 2 iterations. A region of interest (ROI) was designated at the site of maximal accumulation within the mass lesion. The maximal radioactivity of the ROI was determined. FDG uptake was calculated as SUV according to the following formula (constant factor = 10, calibration factor (CF) = 7.40 × 10^6^):

$$ {\mathrm{SUV}}_{\max }=\frac{\mathrm{ROI}\kern0.5em \left(\mathrm{c}\mathrm{p}\mathrm{s}/\mathrm{g}\right)\times \mathrm{constant}\kern0.5em \mathrm{factor}}{\mathrm{injection}\kern0.5em \mathrm{dose}\kern0.5em \left(\mathrm{mCi}\right)/\;\mathrm{bpdy}\kern0.5em \mathrm{weight}\kern0.5em \left(\mathrm{g}\right)\times \left(\mathrm{c}\mathrm{p}\mathrm{s}/\mathrm{mCi}\right)} $$

### Statistical analysis

The data presented were expressed as means ± standard deviation (S.D.) The statistical analysis of SUV_max_ between the groups was performed by Mann-Whitney U test. A *P* value of < 0.05 was considered statistically significant. The SUV_max_ threshold (cut-off value) was determined by the receiver operating characteristic (ROC) analysis. ROC analysis was performed using calculation software IBM SPSS. The performance of the ROC analysis was verified by the ROC-area index Az. The cut-off point was determined by the Youden Index.

## References

[CR1] Bares R, Klever P, Hauptmann S, Hellwig D, Fass J, Cremerius U, Schumpelick V, Mittermayer C, Büll U (1994). F-18 fluorodeoxyglucose PET in vivo evaluation of pancreatic glucose metabolism for detection of pancreatic cancer. Radiology.

[CR2] Beiderwellen KJ, Poeppel TD, Hartung-Knemeyer V, Buchbender C, Kuehl H, Bockisch A, Lauenstein TC (2013). Simultaneous 65Ga-DOTATOC PET/MRI in patients with gastroenteropancreatic neuroendocrine tumors: initial results. Invest Radiol.

[CR3] Berberat P, Freiss H, Kashiwagi M, Berger HG, Büchler MW (1999). Diagnosis and staging of pancreatic cancer by positron emission tomography. World J Surg.

[CR4] Brom M, Oyen WJG, Joosten L, Gotthardt M, Boerman OC (2010). 68Ga-labelled exendin-3, a new agent for the detection of insulinomas with PET. Eur J Nucl Med Mol Imaging.

[CR5] Delbeke D (1999). Oncological applications of FDG-PET imaging. J Nucl Med.

[CR6] Delbeke D, Rose DM, Chapman WC, Pinson CW, Wright JK, Beauchamp RD, Shyr Y, Leach SD (1999). Optimal interpretation of FDG PET in the diagnosis, staging and management of pancreatic carcinoma. J Nucl Med.

[CR7] Delbeke D, Coleman RE, Guiberteau MJ, Brown ML, Royal HD, Siegel BA, Townsend DW, Berland LL, Parker JA, Hubner K, Stabin MG, Zubal G, Kachelriess M, Cronin V, Holbrook S (2006). Procedure guideline for tumor imaging with ^18^F-FDG PET/CT 1.0. J Nucl Med.

[CR8] Eriksson O, Velikyan I, Selvaraju RK, Kandeel F, Johansson L, Antoni G, Eriksson B, Sörensen J, Korsgren O (2014). Detection of metastatic insulinoma by positron emission tomography with [(68)ga]exendin-4-a case report. J Clin Endocrinol Metab.

[CR9] Gabriel M, Decristoforo C, Kendler D, Dobrozemsky G, Heute D, Uprimny C, Kovacs P, Von Guggenberg E, Bale R, Virgolini IJ (2007). 68Ga-DOTA-Tyr3-octreotide PET in neuroendocrine tumors: comparison with somatostatin receptor scintigraphy and CT. J Nucl Med.

[CR10] Herrmann K, Erkan M, Dobritz M, Schuster T, Siveke JT, Beer AJ, Wester HJ, Schmid RM, Friess H, Schwaiger M, Kleeff J, Buck AK (2012). Comparison of 3′-deoxy-3′-[18F] fluorothymidine positron emission tomography (FLT PET) and FDG PET/CT for the detection and characterization of pancreatic tunours. Eur J Nucl Med Mol Imaging.

[CR11] Hong HS, Yun M, Cho A, Choi JY, Kim MJ, Kim KW, Choi YJ, Lee JD (2010). The utility of F-18 FDG PET/CT in the evaluation of pancreatic intraductal papillary mucinous neoplasm. Clin Nucl Med.

[CR12] Imdahl A, Nitzsche E, Krautmann F, Högerle S, Boos S, Einert A, Sontheimer J, Farthmann EH (1999). Evaluation of positron emission tomography with 2-[18F]fluoro-2-deoxy-D-glucose for the differentiation of chronic pancreatitis and pancreatic cancer. Br J Surg.

[CR13] Inokuma T, Tamaki N, Torizuka T, Fujita T, Magata Y, Yonekura Y, Ohshio G, Imamura M, Konishi J (1995). Value of fluorine-18-fluorodeoxyglucose and thallium-201 in the detection of pancreatic cancer. J Nucl Med.

[CR14] Ishizu K, Sadato N, Yonekura Y, Nishizawa S, Magata Y, Tamaki N, Tsuchida T, Okazawa H, Tanaka F, Miyatake S, Ishikawa M, Kikuchi H, Konishi J (1994). Enhanced detection of brain tumors by [18F]fluorodeoxyglucose PET with glucose loading. J Comput Assist Tomogr.

[CR15] Jansson T, Westlin JE, Ahlstrom H, Lija A, Langstrom B, Bergh J (1995). Positron emission tomography studies in patients with locally advanced and/or metastatic breast cancer: A method for early therapy evaluation?. J Clin Oncol.

[CR16] Kamisawa T, Takum K, Anjiki H, Egawa N, Kurata M, Honda G, Tsuruta K (2010). FDG-PET/CT findings of autoimmune pancreatitis. Hepatogastroenterology.

[CR17] Kubota K, Matsuzawa T, Fujiwara T, Ito M, Hatazawa J, Ishiwata K, Iwata R, Ido T (1990). Differential diagnosis of lung tumor with positron emission tomography: a prospective study. J Nucl Med.

[CR18] Lewis JS, Srinivasan A, Schmidt MA, Anderson CJ (1999). In vitro and in vivo evaluation of 64Cu-TETA-Tyr3-octreotate. A new somatostatin analog with improved target tissue uptake. Nucl Med Biol.

[CR19] Mansour JC, Schwartz L, Pandit-Taskar N, D’Angelica M, Fong Y, Larson SM, Brennan MF, Allen PJ (2006). The utility of F-18 fluorodeoxyglucose whole body PET imaging for determining malignancy in cystic lesions of the pancreas. J Gastrointest Surg.

[CR20] Nakamoto Y, Higashi T, Sakahara H, Tamaki N, Kogire M, Imamura M, Konishi J (1999). Contribution of PET in the detection of liver metastasis from pancreatic tumours. Clin Radiol.

[CR21] Nakamoto Y, Higashi T, Sakahara H, Tamaki N, Kogire M, Doi R, Hosotani R, Imamura M, Konishi J (2000). Delayed (18)F-fluoro-2-deoxy-D-glucose positron emission tomography scan for differentiation between malignant and benign lesions in the pancreas. Cancer.

[CR22] Nitzsche EU, Hoegerle S, Mix M, Brink I, Otte A, Moser E, Imdahl A (2002). Non-invasive differentiation of pancreatic lesions: is analysis of FDG kinetics superior to semiquantitative uptake value analysis?. Eur J Nuc Med Mol Imaging.

[CR23] Pedrazzoli S, Sperti C, Pasquali C, Bissoli S, Chierichetti F (2011). Comparison of International Consensus Guidelines versus 18-FDG PET in detecting malignancy of intraductal papillary mucinous neoplasm of the pancreas. Ann Surg.

[CR24] Rempel A, Mathupala SP, Griffin CA, Hawkins AL, Pederson PL (1996). Glucose metabolism in cancer cells: amplification of the gene encoding type II hexokinase. Cancer Res.

[CR25] Sadato N, Tsuchida T, Nakaumra S, Waki A, Uematsu H, Takahashi N, Hayashi N, Yonekura Y, Ishii Y (1998). Non-invasive estimation of the net influx constant using the standardized uptake value for quantification of FDG uptake of tumours. Eur J Nucl Med.

[CR26] Sperti C, Pasquali C, Chierichetti F, Liessi G, Ferlin G, Pedrazzoli S (2001). Value of 18-fluorodeoxyglucose positron emission tomography in the management of patients with cystic tumors of the pancreas. Ann Surg.

[CR27] Sperti C, Pasquali C, Decet G, Chierichetti F, Liessi G, Padrazzoli S (2005). F-18-fluorodeoxyglucose positron emission tomography in differentiating malignant from benign pancreatic cysts: a prospective study. J Gastrointest Surg.

[CR28] Sperti C, Bissoli S, Pasquali C, Frison L, Liessi G, Chierichetti F, Pedrazzoli S (2007). 18-fluorodeoxyglucose positron emission tomography enhances computed tomography diagnosis of malignant intraductal papillary mucinous neoplasm of the pancreas. Ann Surg.

[CR29] Takanami K, Hiraide T, Tsuda M, Nakamura Y, Kaneta T, Takase K, Fukuda H, Takahashi S (2011). Additional value of FDG PET/CT to contrast-enhanced CT in the differentiation between benign and malignant intraductal papillary mucinous neoplasms of the pancreas with mural nodules. Ann Nucl Med.

[CR30] Tann M, Sandrasegaran K, Jennings SG, Skandarajah A, Mchenry L, Schmidt CM (2007). Positron-emission tomography and computed tomography of cystic pancreatic masses. Clin Radiol.

[CR31] Tomimaru Y, Takeda Y, Tatsumi M, Kim T, Kobayashi S, Marubashi S, Eguchi H, Tanemura M, Kitagawa T, Nagano H, Umeshita K, Wakasa K, Doki Y, Mori M (2010). Utility of 2-[18F] fluoro-2-deoxy-D-glucose positron emission tomography in differential diagnosis of benign and malignant intraductal papillary-mucinous neoplasm of the pancreas. Oncol Rep.

[CR32] Wahl RL, Cody RL, Hutchins GD, Mudgett EE (1991). Primary and metastatic breast carcinoma: initial clinical evaluation with PET with the radiolabeled glucose analogue 2-[F-18]-fluoro-2-deoxy D-glucose. Radiology.

[CR33] Wild D, Wicki A, Mansi R, Béhé M, Keil B, Bernhardt P, Christofori G, Ell PJ, Mäcke HR (2010). Exendin-4-based radiopharmaceuticals for glucagonlike peptide-1 receptor PET/CT and SPECT/CT. J Nucl Med.

[CR34] Yoshioka M, Sato T, Furuya T, Shibata S, Andoh H, Asanuma Y, Hatazawa J, Shimosegawa E, Koyama K, Yamamoto Y (2004). Role of positron emission tomography with 2-deoxy-2-[^18^F]fluoro-D-glucose in evaluating the effects of arterial infusion chemotherapy and radiotherapy on pancreatic cancer. J Gastroenterol.

